# Antibiotics Profile, Prevalence of Extended-Spectrum Beta-Lactamase (ESBL), and Multidrug-Resistant *Enterobacteriaceae* from Different Clinical Samples in Khartoum State, Sudan

**DOI:** 10.1155/2020/8898430

**Published:** 2020-10-24

**Authors:** Ehssan H. Moglad

**Affiliations:** ^1^Department of Pharmaceutics, College of Pharmacy, Prince Sattam Bin Abdulaziz University, P.O.Box 173, Alkharj 11942, Saudi Arabia; ^2^Department of Microbiology, Medicinal and Aromatic Plants and Traditional Medicine Research Institute (MAPTMRI), P.O. Box 2404, National Center for Research, Khartoum, Sudan

## Abstract

One of the global requirements for controlling the occurrence of resistance to antimicrobial drugs is to understanding the resistivity profile of various clinical isolates. Therefore, this study aimed to deliver the indication of different resistant profiles of clinically isolated *Enterobacteriaceae* from different sources of samples from Khartoum state, Sudan, and to determine the prevalence rate of extended-spectrum beta-lactamase (ESBL), multidrug-resistant (MDR), extensively drug-resistant (XDR), and pandrug-resistant (PDR) bacteria. A total of 144 Gram-negative bacteria were collected from different sources (vaginal swab, urine, catheter tip, sputum, blood, tracheal aspirate, pus, stool, pleural fluid, and throat swab). Samples were subcultured and identified according to their cultural characteristics and biochemical tests. Antimicrobial susceptibility test was performed for twenty-four antibiotics from eleven categories against all isolated *Enterobacteriaceae* according to the recommendation of Clinical and Laboratory Standards Institute (CLSI). The result showed that out of 144 isolates, *Escherichia coli* and *Klebsiella pneumoniae* were predominant isolates with the percentage of 47.9 and 25%, respectively. The prevalence of ESBL was higher in *K. pneumonia* (38.9%) than *E. coli* (34.8%). All isolated *E. coli* were sensitive to nitrofurantoin and tigecycline. There was a high prevalence of MDR *Enterobacteriaceae*, and only one isolate was XDR, while PDR was zero for all isolated bacteria. Active antimicrobial-resistant (AMR) observation through constant data sharing and management of all stakeholders is crucial to recognize and control the AMR global burden. Also, effective antibiotic stewardship procedures would be applied to limit the unreasonable expenditure of antibiotics in Sudan.

## 1. Introduction

The prevalence of multidrug-resistant bacterial infection has been increasing globally, which is exacerbated due to the scarcity of innovative classes of antibiotics tested clinically during the past 40 years [1]. Bacteria have developed resistant to more powerful antimicrobial agents [2]. In general, bacteria acquired the multidrug resistance either by accumulating several genes each one expressing resistance to one drug in a cell or through the overexpression gene coding for multidrug efflux pumps, flinging a wide range of drugs [3]. Globally, the antimicrobial resistance (AMR) reported that *Escherichia coli* and *Klebsiella* spp. had touched frightening levels of resistance in various parts of the world including resistant to third-generation cephalosporins and carbapenems of up to 54% [4, 5].

Furthermore, information of the resident bacterial etiology and resistance profile is essential to observe any modification that may be happened within time. Therefore, optimum empirical therapy can be achieved by continuous updating of antibiogram [2, 6]. The epidemiological monitoring information can be continuously collected and connected over healthcare settings and nations [7]. Besides, the medicinal community needs full knowledge of the degree of the antimicrobial resistance problem, which can be performed by the association of surveillance data for MDR organisms. Subsequently this phenomenon is prevented by sending the correct information to the public and decision-makers about the expanding danger of MDROs to health community to encourage the wise usage of antimicrobials and other public health procedures [8–10]. Multidrug resistance (MDR) is distinct as developed resistance to at least one agent in three or more antimicrobial groups. Extensive drug resistance (XDR) recognizes as a resistance to at least one agent in all but two or fewer antimicrobial groups; this means bacterial isolates remain sensitive to only one or two groups. Pandrug resistance (PDR) is a resistance to all antibiotics in all antimicrobial groups [7].

From all of the above, worldwide epidemiological observation is critical to comprehensive AMR reaction. Understanding of indigenous and regional AMR is significant for medical choices creation. Still, exploration capability for AMR needs throughout Sudan, and existing AMR records are little, as no full data have been described from the present study area. Therefore, this study aimed to determine the prevalence of *Enterobacteriaceae* in clinical samples and screen the antibiotics profile to the most regularly used antimicrobials and to determine the occurrence rates of extended-spectrum beta-lactamase (ESBL), multidrug resistance (MDR), extensive drug resistance (XDR), and pandrug resistance (PDR).

## 2. Methods

### 2.1. Study Population and Sample Size

This was a cross-sectional study conducted over 6 months from February to July 2018. A total of one hundred and forty-four isolated Gram-negative bacteria from different clinical sample sources (vaginal swab, urine, catheter tip, sputum, blood, tracheal aspirate, pus, stool, pleural fluid, and throat swab) were collected from two main tertiary care referral hospitals in the Khartoum state (Omdurman and Bahri Teaching Hospitals). All Gram-negative isolated bacteria in the microbiology laboratory were included in this study. There are no exclusion criteria.

### 2.2. Identification of Bacterial Isolates

Isolated Gram-negative bacteria were subcultured on nutrient agar and incubated aerobically at 37°C for 24 hours and then identified according to the culture characteristics on Xylose Lysine Deoxycholate agar (XLD), MacConkey agar, and biochemical tests [11].

### 2.3. Antibiotic Susceptibility Test

Various categories of antibiotics were used in this study included cephalosporins, fluoroquinolones, carbapenems, aminoglycosides, penicillins, monobactams, *β*-lactam/*β*-lactamase inhibitor complexes, folate metabolic pathway inhibitors, glycylcyclines, cephamycins, and nitrofuran as recommended for Gram-negative bacteria [7]. The antibiotics used, namely, cefazolin (30 *μ*g), cefuroxime (30 *μ*g), cefotaxime (30 *μ*g), ceftazidime (30 *μ*g), cefepime (30 *μ*g), ciprofloxacin (5 *μ*g), norfloxacin (10 *μ*g), moxifloxacin (5 *μ*g), levofloxacin (5 *μ*g), imipenem (10 *μ*g), meropenem (10 *μ*g), ertapenem (10 *μ*g), tobramycin (10 *μ*g), gentamicin (10 *μ*g), amikacin (30 *μ*g), ampicillin (10 *μ*g), cefoxitin (30 *μ*g), aztreonam (30 *μ*g), amoxicillin-clavulanic acid (30 *μ*g), ampicillin-sulbactam (10/10 *μ*g), trimethoprim/sulfamethoxazole (5/250 *μ*g), piperacillin-tazobactam (100/10 *μ*g), nitrofurantoin (300 *μ*g), and tigecycline (15 *μ*g) were used according to the standard procedures (CLSI). The results were interpreted according to the recommendation of the Clinical Laboratory Standards Institute [12].

### 2.4. Quality Control

For the reliability of study findings, quality control implements measures of performance checks during the entire procedure of the laboratory work. *E. coli* ATCC 25922, *Pseudomonas aeruginosa* ATCC 27853, and *K. pneumoniae* ATCC BAA700603 were used.

### 2.5. Detection of ESBL, MDR, XDR, and PDR

The prevalence of extended-spectrum *β*-lactamase (ESBL) was recognized as the resistance rate against cefotaxime and ceftazidime. MDR, XDR, and PDR were calculated in this study for each isolated bacteria as described by Magiorakos et al. [7].

### 2.6. Statistical Analysis

Data were analyzed using Statistical Package for Social Sciences (SPSS), version 25 (IBM, SPSS Inc., Chicago, IL). Descriptive data were expressed as a percentage. A *P* value of ≤0.05 for the association bacteria and source of samples was considered significant.

## 3. Results

Out of 144 Gram-negative bacterial isolates, *E. coli* and *K. pneumonia* were predominant with percentages 69 (47.9%) and 36 (25%), respectively. However, *Salmonella enterica* was 5 (3.47%), *Shigella* spp. was 1 (0.69%), *Citrobacter freundii* was 1 (0.69%), *Klebsiella oxytoca* was 3 (2.08%), *Enterobacter cloacae* was 1 (0.69%), *Proteus mirabilis* was 3 (2.08%), and *Enterobacter aerogenes* was 2 (1.38%).

Most of the isolated *Enterobacteriaceae* were from urine with a frequency of 51 (35.4%) and 22 (15.37%) for *E. coli* and *K. pneumonia,* respectively. There was a significant association between isolated bacteria and sources of the sample, with *P* value = 0.001 (Table 1). Regarding the resistance rate of antibiotics, *E. coli* revealed the highest resistance rate to ampicillin and aztreonam (66.7 and 40.6%, respectively), while antibiotics from the cephalosporins class exhibited a high resistance rate among *Enterobacteriaceae* bacteria, with the resistance rate for *E. coli* towards ceftazidime (39.1%), and cefotaxime and cefuroxime (37.7%). However, among the drugs belonging to the fluoroquinolones classes, the highest percentage of the resistant rate was obtained by *E. coli* for ciprofloxacin (18.8%) and levofloxacin (17.4%). The lowest resistant rate was obtained for carbapenems drugs with the *E. coli* resistant percentage of 1.4% for imipenem and meropenem. Moreover, all isolated *E. coli* were sensitive towards nitrofurantoin and tigecycline; the resistant rate was zero (Table 2).

There was a high prevalence of MDR bacteria and extended-spectrum *β*-lactamase producer ESBL among *E. coli* and *K. pneumonia* (Table 3). XDR was present only in *E. cloacae* 1(100%) which was sensitive only to amikacin and piperacillin-tazobactam, while PDR was zero for all isolated bacteria.

## 4. Discussion

This study reported the prevalence of Gram-negative *Enterobacteriaceae* as a causative agent for infections, as well as the resistant rate towards various antibiotics belonging to different categories, and the frequency of ESBL, MDR, XDR and PDR were determined.

In the current study, *E. coli* and *K. pneumonia* were the major isolated bacteria from urine samples which mean the leading causative agent for urinary tract infection (UTI). These results entirely agreed with other studies which found that these bacteria were also predominantly causing UTI [13–15]. The study finding stated that *E. coli* and *K. pneumonia* which exhibited a high resistant rate (34.8 and 38.9%, respectively) to cefotaxime and ceftazidime were classified as extended-spectrum *β*-lactamase (ESBL) phenotypes. This ESBL prevalence rate was near to the rate reported in the previous studies from Sudan for *E. coli* and *K. pneumonia* (28.9% and 34.8%) [16], and in agreement with other studies reported the growing prevalence of extended-spectrum beta-lactamase (ESBL) producing isolates from Saudi Arabia ranges from 6% up to 38.5% [17–21].

In general, the increase in the resistance of isolated organisms to penicillin, fluoroquinolones, macrolide, and cephalosporins in this study might be due to the increase in the usage of these antibiotic's classes in the hospital. Also, our study presented the high MDR bacteria, which showed resistance to more than three groups of tested antibiotics. This MDR rate entirely agrees with other studies in Sudan which reported a high prevalence of MDR bacteria [22, 23]. This MDR phenomenon may be due to acquiring of many resistant genes through R plasmid [3]. Furthermore, throughout the latest several decades, the incidence of MDR organisms in hospitals and health centers has increased gradually. So, this study reported the developments of multidrug resistance among *Enterobacteriaceae* and represents an alarming threat of appearance of multidrug-resistant pathogens.

The current study delivers the confirmation of high occurrence of antibiotic-resistant bacteria in urine, tracheal aspirate, and vaginal swab samples. Also, there is a presence of high drug resistance to various antibiotics in *E. coli* and *K. pneumonia* isolates from different samples sources. Some other correlated reports pointed out carelessness from the patient's part, tentative treatment plans, self-prescription, antibiotics misuse, misprescription, and limited information around multidrug-resistant isolates and antimicrobial resistance among clinicians. Nevertheless, the study was conducted in only one state; however, this study will update the records of resistant rates of clinical isolates. Also, it will capture the responsiveness of all hospitals' controlling team in making proper assessments and research to manage the progress of the resistant strain and also help to decrease the alarmingly increasing risk of drug resistance.

## 5. Conclusion

In conclusion, this study highlighted the antibiotics profile and prevalence of ESBL, MDR, XDR, and PDR among *Enterobacteriaceae* from different clinical samples from Khartoum state, Sudan. Therefore, the result of this study may be proof of the urgent need for controlling and managing the development of MDR strain. Moreover, antibiotic stewardship procedures should be applied to limit the illogical use of antibiotics in Sudan.

### 5.1. Limitations

Our perspective cross-sectional study has some limitations that should be recognized, which it was conducted in one state (Sudan consists of 18 states), which could not reflect the epidemiology of different states or different geographical areas.

## Figures and Tables

**Table 1:** Distribution and relationship between isolates and source of samples.

Gram-negative bacteria	Sample sources										Total *N* (%)
	Vaginal swab	Urine	Catheter tip	Sputum	Blood	Tracheal aspirate	Pus	Stool	Pleural fluid	Throat swab	
*E. coli*	11	51	0	0	0	4	3	0	0	0	69 (47.9)
*K. pneumonia*	5	22	1	4	1	1	2	0	0	0	36 (25)
*Ps. aeruginosa*	0	3	0	0	1	10	0	0	0	1	15 (10.4)
*S. enterica*	0	0	0	0	1	0	0	4	0	0	5 (3.5)
*Shigella* spp.	0	0	0	0	0	0	0	1	0	0	1(0.69)
*P. fluorescence*	0	0	0	0	1	0	0	0	0	0	1(0.69)
*C. freundii*	0	1	0	0	0	0	0	0	0	0	1(0.69)
*Acinetobacter lwoffii*	0	0	0	0	1	0	0	0	0	0	1(0.69)
*K. oxytoca*	0	3	0	0	0	0	0	0	0	0	3 (2.1)
*E. cloacae*	0	0	0	0	0	1	0	0	0	0	1(0.69)
*P. mirabilis*	0	2	0	0	0	0	0	1	0	0	3 (2.1)
*Providencia stuartii*	0	1	0	0	0	0	0	0	0	0	1(0.69)
*G. vaginalis*	0	0	0	0	0	0	0	0	1	0	1(0.69)
*E. aerogenes*	1	1	0	0	0	0	0	0	0	0	2 (1.4)
*A. baumannii*	0	1	0	0	0	0	0	0	0	0	1(0.69)
*M. morganii*	0	1	0	0	0	0	0	0	0	0	1(0.69)
*H. influenzae*	1	0	0	0	0	0	0	0	0	0	1(0.69)
*S. maltophilia*	0	1	0	0	0	0	0	0	0	0	1(0.69)
Total											
N (%)	18 (12.5)	87 (60.4)	1(0.69)	4 (2.8)	5 (3.5)	16 (11.1)	5 (3.5)	6 (4.2)	1 (0.69)	1 (0.69)	144 (100)
^*∗*^ *P* value	0.001										

Note: *Escherichia coli*, *Klebsiella pneumoniae*, *Pseudomonas aeruginosa*, *Salmonella enterica*, *Shigella* spp., *Pseudomonas fluorescens*, *Citrobacter freundii*, *Acinetobacter lwoffii*, *Klebsiella oxytoca*, *Enterobacter cloacae*, *Proteus mirabilis*, *Providencia stuartii*, *Gardnerella vaginalis*, *Enterobacter aerogenes*, *Acinetobacter baumannii*, *Morganella morganii*, *Haemophillus influenzae* group 11, and *Stenotrophomonas maltophilia*. The relationship between the isolates and sources of samples was significant, ^*∗*^*P* value < 0.05.

**Table 2:** Percentage of antimicrobial resistance patterns of *Enterobacteriaceae* isolated from different clinical samples.

Name	N	CIP	NOR	LVX	MXF	CTX	CZ	FEP	CXM	CAZ	IPM	MEM	ETP	TM	GM	AN

*E. coli*	69	18.8	10.1	17.4	7.2	37.7	34.8	36.2	37.7	39.1	1.4	1.4	2.9	15.9	15.9	1.4
*K. pneumonia*	36	8.3	5.6	5.6	11.1	47.2	38.9	41.7	50.0	47.2	5.6	8.3	11.1	16.7	5.6	8.3
*S. enterica*	5	0	0	0	0	0	0	0	NT	0	0	0	0	NT	NT	0
*Shigella* spp.	1	0	0	0	0	0.7	NT	0	NT	0	0	0	0	NT	NT	NT
*C. freundii*	1	0	0	0	0	0	NT	0	0	0	0	0	0	0	0	0
*K. oxytoca*	3	33.3	33.3	33.3	0.0	66.7	33.3	66.7	66.7	33.3	0.0	33.3	66.7	33.3	33.3	33.3
*E. cloacae*	1	100	NT	100	100	100	NT	100	100	100	100	100	100	100	100	0
*P. mirabilis*	3	0.0	0.0	0.0	0.0	0.0	0.0	33.3	0.0	33.3	0.0	33.3	33.3	0.0	0.0	0.0
*E. aerogenes*	2	0	0	0	0	50	NT	0	100	0	50	0	0	0	0	0

Name	N	AMC	SAM	TZP	FOX	AM	FT	TGC	SXT	ATM						
*E. coli*	69	10.1	27.5	2.9	7.2	66.7	0.0	0.0	33.3	40.6						
*K. pneumonia*	36	13.9	27.8	5.6	16.7	94.4	22.2	8.3	36.1	50.0						
*S. enterica*	5	0	20	0	NT	20	NT	0	40	0						
*Shigella* spp.	1	0	0	0	NT	100	NT	0	0	100						
*C. freundii*	1	0	0	0	100	0	0	0	0	0						
*K. oxytoca*	3	33.3	66.7	0.0	66.7	100	0.0	33.3	66.7	33.3						
*E. cloacae*	1	100	100	0	100	100	NT	100	100	100						
*P. mirabilis*	3	33.3	33.3	33.3	0.0	33.3	66.7	0.0	33.3	66.7						
*E. aerogenes*	2	100	100	0	100	100	0	0	0	0						

Note: *N* = total number of tested bacteria, CIP = ciprofloxacin, NOR = norfloxacin, MXF = moxifloxacin, LVX = levofloxacin, CTX = cefotaxime, CZ = cefazolin, FEP = cefepime, CXM = cefuroxime, CAZ = ceftazidime, IPM = imipenem, MEM = meropenem, ETP = ertapenem, TM = tobramycin, GM = gentamicin, AN = amikacin, AMC = amoxicillin-clavulanate, SAM = ampicillin-sulbactam, TZP = piperacillin-tazobactam, FOX = cefoxitin, AM = ampicillin, FT = nitrofurantoin, TGC = tigecycline, SXT = trimethoprim-sulfamethoxazole, ATM = aztreonam, NT = not tested.

**Table 3:** The prevalence of MDR and ESBL among *Enterobacteriaceae*.

	Number and percentage of isolated		MDR		ESBL	
			Yes	No	Yes	No
*E. coli*	*N*	69	34	35	24	45
	%	100	49.3	50.7	34.8	65.2
*K. pneumonia*	*N*	36	21	15	14	22
	%	100	58.3	41.7	38.9	61.1
*S. enterica*	*N*	5	1	4	0	5
	%	100	20	80	0	100
*Shigella* spp.	*N*	1	1	0	0	1
	%	100	100	0.0	0.0	100
*K. oxytoca*	*N*	3	3	0	1	2
	%	100	100	0.0	33.3	66.7
*P. mirabilis*	*N*	3	1	2	0	3
	%	100	33.3	66.7	0.0	100
*E. aerogenes*	*N*	2	2	0	0	2
	%	100	100	0.0	0.0	100
*E. cloacae*	*N*	1	1	0	1	0
	%	100	100	0	100	0
*P* value			0.001		0.001	

Note: MDR = multidrug-resistant bacteria, ESBL = extended-spectrum *β*-lactamase producer.

## Data Availability

All data generated or analyzed during this study are included within this article.

## Abbreviations

ESBL: Extended-spectrum beta-lactamase
MDR: Multidrug-resistant
XRD: Extensively drug-resistant
PDR: Pandrug-resistant
CLSI: Clinical and Laboratory Standards Institute
AMR: Antimicrobial-resistant
XLD: Xylose Lysine Deoxycholate Agar
ATCC: American Type Culture Collection.
